# PD-L1 Expression on Circulating Tumour-Derived Microvesicles as a Complementary Tool for Stratification of High-Grade Serous Ovarian Cancer Patients

**DOI:** 10.3390/cancers13205200

**Published:** 2021-10-16

**Authors:** Alessandra Battaglia, Alessia Piermattei, Alexia Buzzonetti, Tina Pasciuto, Nicole Zampetti, Marco Fossati, Giuseppe Angelico, Valentina Iacobelli, Camilla Nero, Veronica Iannucci, Giovanni Scambia, Anna Fagotti, Andrea Fattorossi

**Affiliations:** 1 Department of Life Science and Public Health, Università Cattolica del Sacro Cuore, 00168 Rome, Italy; camilla.nero@policlinicogemelli.it (C.N.); giovanni.scambia@policlinicogemelli.it (G.S.); anna.fagotti@policlinicogemelli.it (A.F.); 2 Department of Women, Children and Public Health Sciences, Fondazione Policlinico Universitario Agostino Gemelli IRCCS, 00168 Rome, Italy; alessia.piermattei@policlinicogemelli.it (A.P.); alexia.buzzonetti@policlinicogemelli.it (A.B.); tina.pasciuto@policlinicogemelli.it (T.P.); nicole.zampetti@policlinicogemelli.it (N.Z.); marco.fossati@policlinicogemelli.it (M.F.); giuseppe.angelico@aoec.it (G.A.); vale.iacobelli@gmail.com (V.I.); veronica.iannucci@policlinicogemelli.it (V.I.); andrea.fattorossi@policlinicogemelli.it (A.F.)

**Keywords:** translational research, microvesicles, biomarkers, PD-L1, immunohistochemistry, ovarian cancer, precision medicine, flow cytometry

## Abstract

**Simple Summary:**

PD-1/PD-L1 axis blockade immunotherapy leverages on assessment of tumour PD-L1 status by immunohistochemistry for identifying cancer patients’ who would benefit most from this therapy. Because some PD-L1 negative cancer patients also benefit from the treatment, it has been postulated that spatial heterogeneity of PD-L1 expression in tumour tissue may reduce the predictive value of the test. Here we demonstrate for the first time the presence of PD-L1+ circulating tumor–derived microvesicles in the blood stream of high grade serous ovarian cancer patients and propose the assessment of tumor-derived circulating PD-L1+ microvesicles to be inserted in predictive algorithms for complementing tumour PD-L1 status.

**Abstract:**

Background: Ovarian cancer (OC) has recently attracted attention for the use of PD-1/PD-L1 axis blocking agents, with durable activity reported only in a subset of patients. The most used biomarker for sensitivity to the PD-1/PD-L1 axis blockade is tumour PD-L1 status by immunohistochemistry. However, patient stratification using this method suffers from intrinsic heterogeneity of OC, likely contributing to the unsatisfactory results obtained so far. Cells communicate with each other by releasing microvesicles (MVs) that carry parental cell surface features. Thus, we hypothesised that PD-L1^+^ tumour cells (TC) and infiltrating PD-L1^+^ leukocytes should shed MVs carrying surface PD-L1 that may serve as a proxy for the whole tumour PD-L1 status. Results: We showed for the first time the presence of measurable amounts of TC- and leukocyte-derived PD-L1^+^ MVs (range: 1.4–178.8 MVs/μL and 6.2–504.8 MVs/μL, respectively) in the plasma of high-grade serous OC (HGSOC) patients (*n* = 63), using a sensitive flow cytometry platform. The concentration of PD-L1^+^ MVs of either origin did not associate with the PD-L1 status of TCs and leukocytes in the tumour biopsies, suggesting that the circulating PD-L1^+^ MVs also included ones from locations not selected for immunohistochemistry analysis and represented the PD-L1 status of the whole tumour mass. In this study, we also describe the serendipitous discovery of circulating PD-L1^+^ MVs of platelet origin (10.3–2409.6 MVs/μL). Conclusions: The enumeration of circulating PD-L1^+^ MVs in HGSOC patients may provide a novel direction for assessing the tumour PD-L1 status and contribute to HGSOC patient stratification for immunotherapy interventions. The presence of circulating PD-L1^+^ MVs of platelet origin, a finding not yet reported in HGSOC patients, warrants further studies.

## 1. Introduction

Ovarian cancer (OC) is the leading cause of death among gynaecological cancers in developed countries [1]. The most common histotype is high-grade serous carcinoma (HGSOC), and more than two-thirds of patients are diagnosed at an advanced stage. The mainstay of treatment is surgical debulking and platinum-based chemotherapy [2,3,4]. In the last ten years, target therapies, such as vascular endothelial growth factor (VEGF) and poly(ADP-ribose) polymerase (PARP) inhibitors, have been introduced in the therapeutic algorithm of OC, providing remarkable prognostic improvements [5]. Nevertheless, up to 80% of advanced OC patients relapse within 24 months. Thus, the best therapeutic option for each patient remains a major issue to be addressed [6,7,8].

Great interest has arisen in investigating the blockade of programmed cell death protein-1 (PD-1) and its ligand (PD-L1) in a variety of cancers, including OC [9]. This immunotherapeutic approach requires that tumour cells and/or tumour-infiltrating immune cells express PD-L1. Despite the fact that ≥50% of advanced stage OCs express PD-L1 [10], data from clinical trials have shown unsatisfactory response rates [11,12,13,14,15,16]. Nevertheless, other trials have been designed and are currently ongoing (e.g., the ENGOT-ov46/AGO/DUO-O, ENGOT-OV43/KEYLYNK-001, and ATHENA phase III clinical studies).

The assessment of PD-1/PD-L1 expression in tumour biopsies is currently used as a biomarker for sensitivity to PD-1/PD-L1 axis blockade treatment in a variety of tumours. However, since some PD-L1 negative patients benefit from targeting the PD-1/PD-L1 axis [17,18,19,20], it has been postulated that the spatial heterogeneity of PD-L1 expression in tumour tissue, which has been conclusively reported in previous studies [21,22,23], makes PD-L1 scoring in single tumour biopsies not representative for the entire tumour PD-L1 status.

In both physiological and pathological settings, cells communicate with each other by releasing extracellular vesicles (EVs). EVs principally include three populations distinguishable by size, composition, and biogenesis: exosomes (50–100 nM size), microparticles, also called microvesicles (MVs) (100 nM to 1 μM size), and apoptotic bodies (≥1 μM) [24]. MVs are easily accessible in patients’ blood, and, being generated by outward budding and fission of the plasma membrane [25,26], they express the phenotype of the cells from which they originate. This allows traceability of the MVs’ origin through surface immunostaining and multicolour flow cytometry, making them ideal candidates for “liquid biopsies”.

In the context of cancer biology, evidence is rising that tumour cells, including OC cells, secrete EVs that carry a cargo which is typical of the cell of origin, which facilitates the spread of cancer by priming distant areas of the body to be cancer cell-friendly [27,28,29]. In the OC setting, several studies have pointed to the relevance of EVs present in patients’ plasma, serum, and ascites. The cargo of tumour-derived EVs in the ascites of OC patients has been reported to contain molecules involved in extracellular matrix degradation, thus facilitating tumour cell invasion and metastasis [30]. Circulating EVs in OC patients have been reported to contain the enzyme tyrosine receptor kinase B, related to OC progression [31]. OC effusion-derived EVs have been demonstrated to contain a particular microRNA profile that was related to disease outcome [32]. Collectively, these studies indicate that OC cells release pro-tumoral EVs to promote their survival and spread. Consistently, circulating EVs have been proposed as a prognostic indicator in OC patients [33,34].

In this study, we evaluated the presence of circulating tumour-derived PD-L1^+^ MVs and their association with tumour PD-L1 status in a prospective cohort of HGSOC patients.

## 2. Results

### 2.1. Study Population

The study population consisted of 63 patients with histologically confirmed diagnosis of HGSOC FIGO stage IIIB-C and IVA-B [23] from April 2019 to November 2019 at the division of Gynaecologic Oncology, Fondazione Policlinico Universitario A. Gemelli IRCCS, Rome, Italy. Baseline clinico-pathological characteristics are summarised in Table 1.

Twenty-six patients (41.3%) were referred for primary debulking surgery (PDS) and adjuvant chemotherapy (PDS+ACT). When PDS was deemed not feasible, patients (*n* = 37; 58.7%) were referred for neoadjuvant chemotherapy (NACT) + interval debulking surgery (IDS). 

**Figure 1:**
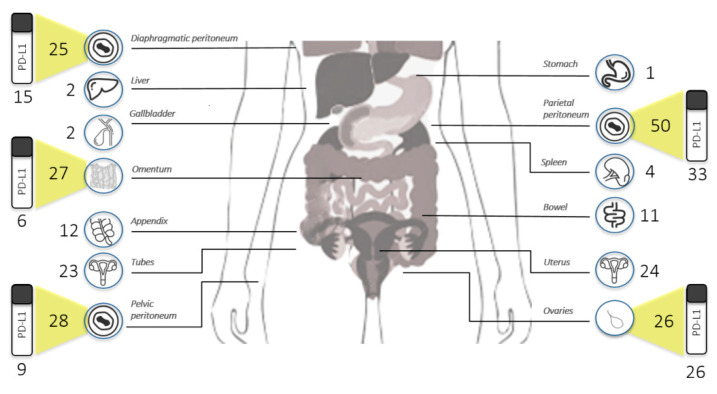
Sites of primary ovarian cancer and metastasis biopsies collected throughout the study.

Tissue biopsies obtained and subsequent IHC PD-L1 analysis are depicted in Figure 1. Metastatic biopsies were available for all patients enrolled. A primary tumour sample was available only for patients undergoing PDS. Thus, 89 FFPE tissue blocks were examined.

### 2.2. Histological Features and Evaluation of PD-L1 Expression 

PD-L1 expression was evaluated in 89 tissue biopsies from primary (*n* = 26) and metastatic (*n* = 63) HGSOC tissue biopsies. The median size of tumour tissue on slides was 16 mm (range: 1–30 mM) for primary tumours and 6 mm (range: 1–24 mM) for metastases. In detail, metastatic tumour samples included 33 parietal peritoneal biopsies, nine pelvic peritoneal, 15 diaphragmatic peritoneal, and six omental biopsies. No difference in terms of PD-L1 expression on tumour cells (TCs) and immune cells present (ICP) was observed between patients stratified according to type of surgery (Table 2).

**Figure 2:**
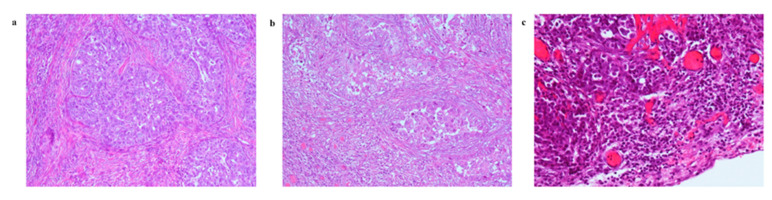
Representative examples for haematoxylin and eosin stain of tumour-infiltrating lymphocytes (TILs) in primary and metastatic ovarian tumour samples. (**a**) Low-grade density (TILs ≤ 10%) (10× magnification); (**b**) intermediate-grade density (10% < TILs < 40%) (10× magnification); (**c**) high-grade density (TILs ≥ 40%) (20× magnification).

The presence of TCs and clusters of immune cells characterised all tumour lesions. Immune cells in the tumour microenvironment mainly consisted of intraepithelial TILs, plasma cells, and eosinophils (in 3.2% of all cases). Histiocytic cells were observed in one metastatic biopsy. The evaluation of TILs in metastatic biopsies showed that: 47/63 (74.6%) patients had TIL levels ≤ 10%; 12/63 (19.0%) patients had 10% < TILs < 40%; and 4/63 (6.3%) patients had TIL levels ≥ 40.0% (representative tumour samples showing the variable amounts of TILs are depicted in Figure 2). 

In order to verify an association of TIL levels in primary tumour and metastasis biopsies, we then compared TIL frequencies for 26 patients with both primary tumour and metastasis biopsies. In detail, primary tumour and metastasis biopsies showed levels of TILs ≤ 10% in 20/26 (76.9%) and 16/26 (61.5%) cases, respectively. Primary tumour and metastasis biopsies showed 10% < TILs < 40% in 4/26 (15.4%) and 7/26 (26.9%) cases, respectively. Finally, primary tumour and metastasis biopsies showed levels of TILs ≥ 40% in 2/26 (7.7%) and 3/26 (11.5%) cases, respectively. Overall, TIL levels were similar between primary tumour and metastasis biopsies in 61.5% of patients. 

**Figure 3:**
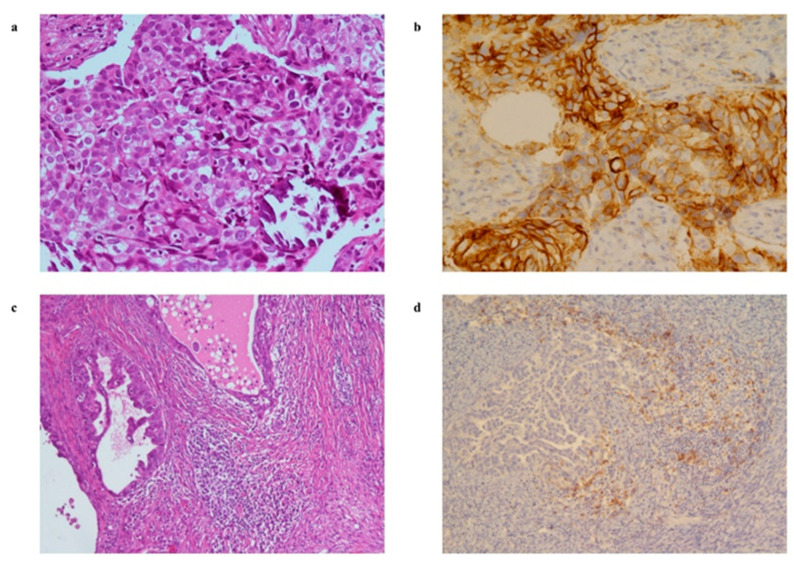
Representative examples for histological and immunohistochemical staining of PD-L1 on TCs (upper panels) and tumour-infiltrating inflammatory/immune cells (lower panels) on HGSOC tumour biopsies. (**a**) Haematoxylin and eosin stain of metastatic tumour biopsy (20× magnification); (**b**) immunohistochemical analysis showing 85% of tumour cells stained positively for PD-L1 in metastatic tumour biopsy (20× magnification); (**c**) haematoxylin and eosin stain of primary ovarian tumour (10× magnification); (**d**) clusters of immune cells with PD-L1 positive cytoplasmic +/− membrane staining, in primary ovarian tumour (10× magnification).

The PD-L1 status evaluated by IHC is depicted in Figure 3. PD-L1 staining showed a similar pattern in both TCs and ICPs in primary tumours and metastatic samples. In particular, TCs showed a complete medium/strong membranous staining (Figure 3b), and ICPs, mostly consisting of TILs, were found organised in aggregates and exhibited a membrane, cytoplasm, dense, or punctuated PD-L1 staining (Figure 3d). The median percentage of PD-L1-expressing TCs in primary (26 samples) and metastatic (63 samples) tumour biopsies was 1% (min%–max%: 0–30) and 1% (min%–max%: 0–85), respectively. 

**Figure 4:**
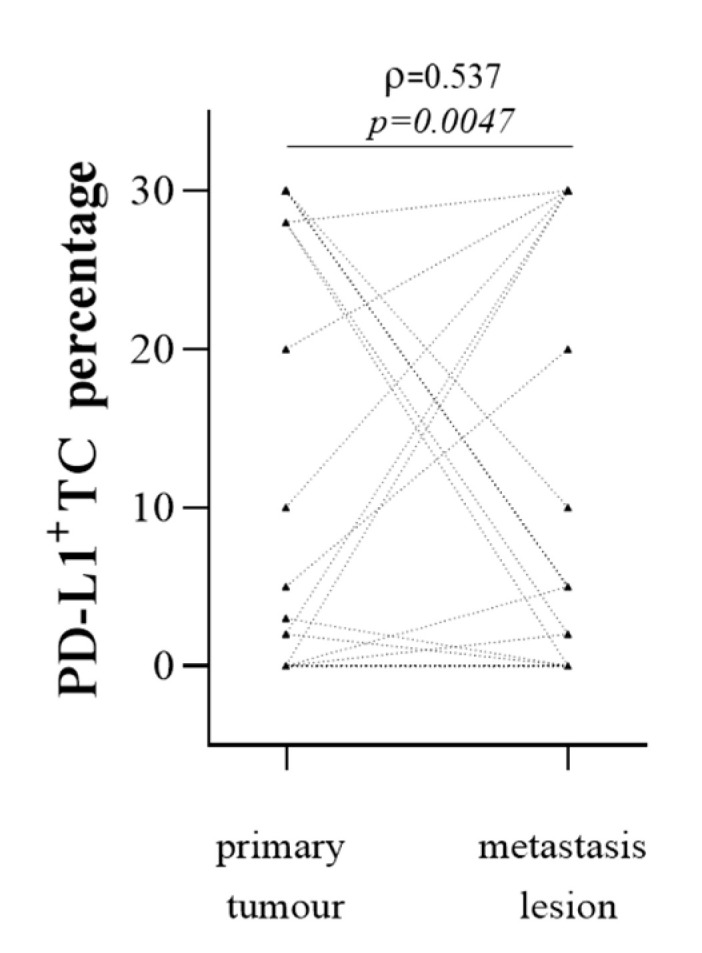
PD-L1^+^ TC frequency in primary tumour and metastatic biopsies by IHC assay. Black triangles indicate percentages of PD-L1^+^ TCs observed in primary tumours (left series) and metastasis biopsies (right series). Dotted black lines indicate primary tumours and metastasis lesions obtained from the same patient. ρ, Spearman correlation coefficient.

The correlation in the extent of PD-L1-expressing TCs between the primary and the metastatic tumour sites was then verified in the 26 patients with both primary tumour and metastasis lesions with a coefficient of correlation rho = 0.54 (Figure 4). 

We then verified the association in the expression of PD-L1 on TCs according to the 1% binary cut-off (see M&M) in patients with both primary and metastatic tumour biopsies. Results showed a significant association in the PD-L1 status of TCs in the primary tumour and the metastatic districts (*p* = 0.006 by chi-squared test, Table 3). However, dissimilar PD-L1 status between the two districts was observed in 6/26 (23.1%) patients studied (Table 3).

As reported above, ICPs were always identified in tumour samples. PD-L1-expressing ICPs were found in 30.8% of primary tumour biopsies and in 23.8% of metastasis biopsies (Table 2). We next verified the association in PD-L1 expression on ICPs according to the 1% binary cut-off (see M&M) in patients with both primary tumour and metastasis biopsies. Results showed no association in the PD-L1 status of ICPs in the primary tumour and the metastatic districts (*p* = 0.272 by chi-squared test) (Table 3). 

No statistically significant association of PD-L1 status in patients with FIGO stage IIIB–IIIC versus IVA–IVB was observed, neither in terms of TCs nor ICPs.

### 2.3. Identification of Tumour- and Leukocyte-Derived PD-L1-Expressing MVs by Flow Cytometry

**Figure 5:**
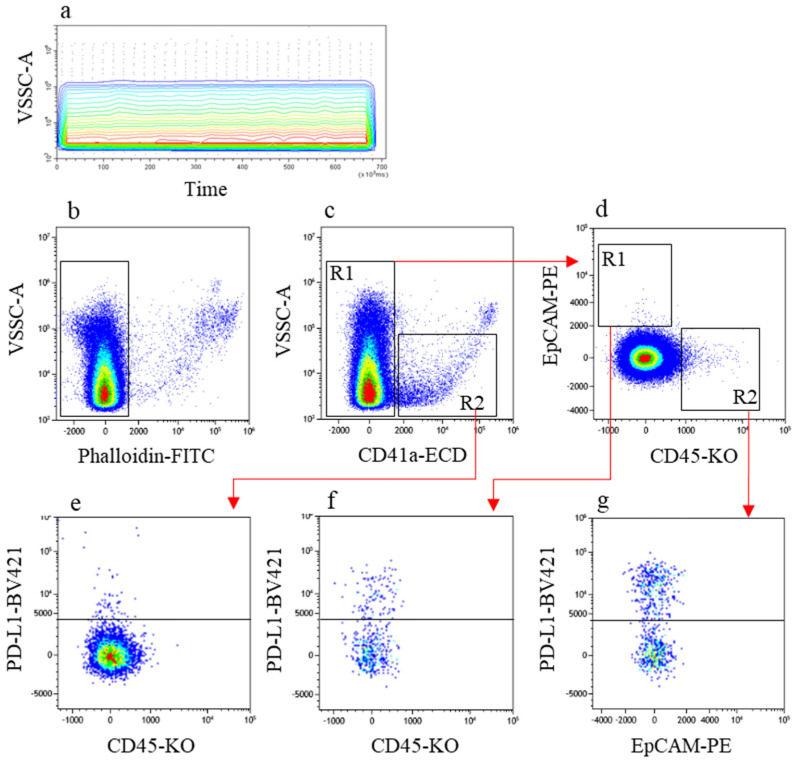
Flow cytometry analytical gating strategy used to identify MVs. Manual analysis of specific MV populations was performed with visual help of the flow cytometer software. (**a**) Flow stability verification on VSSC-A vs. parameter time; (**b**) unwanted, non-intact phalloidin-positive MVs were removed. (**c**) In this cleaned MV population, a PLT-derived MV-free region (R1) and a PLT-derived MV region (R2) were recognised. (**d**) MVs of tumour (R1) and leukocyte (R2) origin were recognised on bivariate dot plot. There were no double-stained MVs, indicating absence of coincident events (swarming recognition). Finally, PD-L1-expressing TC-derived MVs (**f**), PD-L1-expressing leuko-derived MVs (**g**), and PD-L1-expressing PLT-derived MVs (**e**) were recognised and used for analysis.

Figure 5 exemplifies the gating strategy to identify and quantify the MV populations of interest. First, flow stability was verified (Figure 5a), and intact MVs were identified in a Phalloidin/VSSC-A plot (Figure 5b). Figure 5c depicts the CD41a/VSSC-A profile. Region R1 excluded platelets (PLT) and PLT-derived MVs and served to generate the dual colour CD45/EpCAM plot (Figure 5d). Figure 5d served to identify EpCAM^+^ CD45^−^ MVs (hereafter referred to as TC-derived MVs, R1) and CD45^+^ EpCAM^−^ MVs (hereafter referred to as leuko-derived MVs, R2). PD-L1 expression on TC-derived MVs was then measured in the CD45 versus PD-L1 plot (Figure 5f). PD-L1 expression on leuko-derived MVs was then measured in the EpCAM versus PD-L1 plot (Figure 5g).

In a different tumour setting, Rolfes et al. [36] demonstrated that PLTs express PD-L1. Thus, we explored whether PLT-derived MVs expressed PD-L1. Region R2 in Figure 5c included all MVs of PLT origin and served to enumerate PLT-derived MVs and PD-L1^+^ PLT-derived MVs (Figure 5e).

Results are summarised in Table 4. All plasma samples contained both TC-derived and leuko-derived MVs, and sizeable amounts of MVs of either origin expressed PD-L1. Stratification of patients according to FIGO stage (IIIB–IIIC and IVA–IVB) did not recognise patients with different capacities to generate MVs of either origin. Likewise, PD-L1 expression on TC- and on leuko-derived MVs was similar between FIGO stages. A large number of PLT-derived MVs were present in all plasma samples, and a measurable amount of them expressed PD-L1 with no relationship to FIGO stage.

### 2.4. Evaluating Association between PD-L1 Status in Tumour Biopsies and PD-L1 Expressing TC- and Leuko-derived MVs 

**Figure 6:**
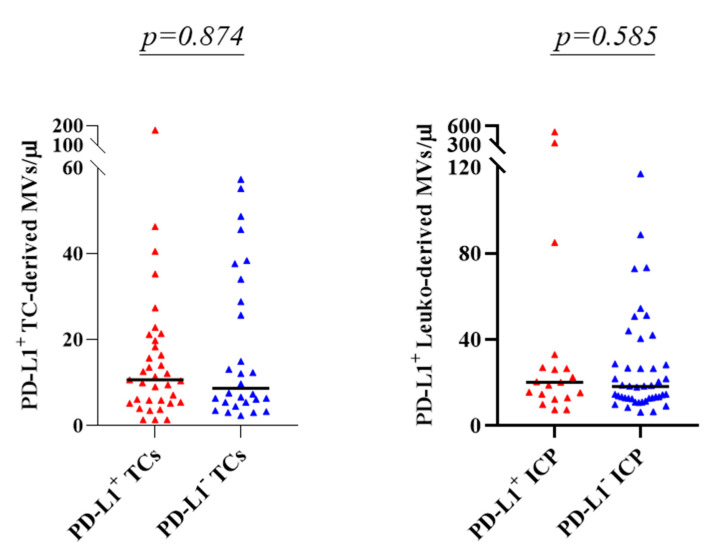
Distribution of plasma levels of PD-L1^+^ MVs in patients stratified according to PD-L1 scoring by IHC. Left panel: plasma levels of PD-L1^+^ TC-derived MVs in patients with PD-L1^+^ TCs (*n* = 35) in any biopsy and patients with PD-L1^−^ TCs (*n* = 28). Right panel: plasma levels of PD-L1^+^ leuko-derived MVs in patients with PD-L1^+^ ICPs in any biopsy (*n* = 19) and patients with PD-L1^−^ ICPs (*n* = 44). Horizontal bars indicate median values. p: *p*-value by Mann–Whitney test.

The relationship between plasma levels of PD-L1^+^ MVs derived from TCs and from leukocytes and PD-L1 expression on TCs and ICPs in tissue samples was tested. Patients were stratified for PD-L1 expression on TCs and ICPs in tissue samples according to an overall IHC assessment. Patients were considered positive for PD-L1 expression on TCs when at least one biopsy scored positive for PD-L1. As shown in Figure 6 (left panel), plasma levels of PD-L1^+^ TC-derived MVs equally distributed in the two groups (*p* = 0.874 by Mann–Whitney test). 

An analogous strategy was used to investigate the association between PD-L1-expressing leuko-derived MVs and overall IHC assessment (see above) of PD-L1-expressing ICPs in the tumour. As shown in Figure 6 (right panel), plasma levels of PD-L1^+^ leuko-derived MVs equally distributed in the two groups (*p* = 0.585 by Mann–Whitney test). 

## 3. Discussion

In the present study, we explored the presence of circulating tumour-derived PD-L1^+^ MVs and their association with tumour PD-L1 status by IHC in HGSOC patients.

By quantitative flow cytometry, we showed for the first time that OC cells and leukocytes release PD-L1^+^ MVs into the blood stream.

IHC analysis showed that 50.0% of metastasis biopsies contained PD-L1^+^ TCs ≥ 1%, whereas only around 40% of primary tumour biopsies contained PD-L1^+^ TCs ≥ 1%, suggestive of OC heterogeneity between different tumour sites. Consistently, among patients in which both primary tumour and metastasis biopsies were available for the TC PD-L1 status assessment, an association in PD-L1 scores between biopsies in only around 75% of patients was shown. Similar results were obtained for the ICP PD-L1 status. These results are not surprising; the divergent PD-L1 expressions in different tumour sites due to intrinsic tumour heterogeneity has already been highlighted in a variety of malignancies [21,22,23]. Similar observations have been reported in the OC setting; evaluation of PD-L1 expression in tumour biopsies falls short of reflecting the PD-L1 status of the entire tumour unless several lesions are scored [37,38]. The discordance rate of the estimated PD-L1 score between specimens from different tumour sites highlights the difficulty of representing PD-L1 status with a single test value and is relevant for clinical decision-making when anti-PD-L1/PD-1 blocking agents are a treatment option. 

The association between plasma levels of PD-L1^+^ MVs of tumour or leukocyte origin and PD-L1 status in the tumour biopsies was investigated. No difference in plasma concentration of TC-derived PD-L1^+^ MVs between patients scoring negative and patients scoring positive for PD-L1^+^ TCs by IHC was demonstrated. This finding is similar to a previous study in NSCLC patients that likewise did not find agreement between PD-L1-expressing TCs in tumour biopsies and circulating PD-L1-expressing exosomes [39]. In agreement with Li et al. [39], we conclude that circulating PD-L1^+^ MVs in patients classified as PD-L1^−^ by IHC indicate the presence of PD-L1^+^ TCs at tumour sites not explored by IHC testing, highlighting that PD-L1 scoring by IHC is biased by heterogeneous PD-L1 expression in the tumour. That OC heterogeneity is an issue in determining PD-L1 status is in line with the incomplete agreement in PD-L1 expression between primary and metastatic sites found in our research. Thus, MV assessment may solve the issue of spatial tumour heterogeneity and contribute to an accurate representation of the actual PD-L1 status of the whole tumour mass, since circulating TC-derived PD-L1^+^ MVs are representative of PD-L1^+^ TCs at any tumour location. In analogy with earlier studies in different tumour settings [40,41], future studies aimed at optimising OC patient stratification for anti-PD-1/anti-PD-L1 treatments might benefit from measuring circulating TC-derived PD-L1^+^ MVs. Noteworthily, complementing PD-L1 assessment by IHC with quantification of plasma levels of PD-L1^+^ TC-derived MVs might also contribute to solve the still controversial issue of the prognostic significance of PD-L1 expression on TCs in OC patients—either high expression indicating poor prognosis [42,43], favourable prognosis [10,44,45], or irrelevancy [37,38].

We did not find agreement between leuko-derived PD-L1^+^ MVs and PD-L1 expression on leukocytes in the biopsies. We are not aware of similar studies, either in OC or in other tumour settings. In analogy to TC-derived PD-L1^+^ MVs, we suggest that this disagreement can be explained by the release into the circulation of leuko-derived PD-L1^+^ MVs from tumour locales that were not sampled for IHC assay. Supporting this view, we found a heterogeneous PD-L1 expression on immune cells from different tumour locales by IHC, which is in accord with earlier studies [38,44]. An alternative, non-mutually exclusive explanation is that PD-L1^+^ immune cells from non-neoplastic tissues contributed to the global leuko-derived PD-L1^+^ MVs pool. In that regard, an early report described the presence of circulating PD-L1^+^ leukocyte populations in NSCLC patients [46]. 

Early studies have focused on measuring tumour-derived MV levels in the plasma of cancer patients using a methodology similar to the one we used, i.e., surface profiling by antibodies specific for cancer cell membrane antigens and flow cytometry, and have reported that plasma levels of TC-derived MVs are increased in advanced tumour stages in breast [47] and hepatocellular carcinomas [48]. In the present study, we did not find any significant difference between MV numbers in OC patients with different FIGO stages, i.e., IIIB–IIIC vs. IVA–IVB. Although the discrepancy between early and present results may mirror differences in the modality of MV shedding in OC vs. breast and hepatocellular carcinoma, a more likely explanation is that in the OC setting the IIIB–IIIC and IVA–IVB FIGO stages are both advanced stages. Further studies including patients with earlier OC FIGO stages are needed to solve this issue.

An unexpected finding in the course of this study was the expression of PD-L1 on circulating PLT-derived MVs. PLTs carrying PD-L1 have been observed in healthy smokers and in patients with various types of malignancies [36,49] and have been interpreted as a consequence of PLT activation [49]. Activated PLTs also show enhanced MV release [50]. Thus, these two concomitant factors may have contributed to the large number of PLT-derived PD-L1^+^ MVs we found. Because PD-L1-expressing PLTs were suggested to add to the array of mechanisms employed by tumour cells evading the immune attack [49], we may also infer that PLT-derived PD-L1^+^ MVs may be endowed with a similar pro-tumour activity. Thus, the presence of circulating PLT-derived PD-L1^+^ MVs introduces them as potentially novel and non-invasive biomarkers in OC with yet unexplored implications and deserves additional studies.

A limitation of this study was the impossibility of correlating the PD-L1 status evaluated by the combination of IHC scoring on tumour biopsies and of flow cytometry assessment of circulating PD-L1^+^ MVs with response to immunotherapy, since only standard chemotherapeutic regimens were adopted in our cohort.

## 4. Materials and Methods

### 4.1. Study Population and Design

This is a not-for-profit, observational, single centre study co-financed by Roche SpA. Patients with histologically proven advanced HGSOC with no previous chemotherapy were eligible. Exclusion criteria included history of concomitant or previous malignancy in the last 5 years. Study data were collected and managed using REDCap (Research Electronic Data Capture) electronic data capture tools hosted at (https://redcap-irccs.policlinicogemelli.it/, accessed on 21 June 2018) [51,52]. Only staff officially registered as study investigators or data managers received a user login to access the REDCap web platform to enter/manage data.

### 4.2. Tissue Samples

Primary tumour and metastasis biopsies were obtained before any treatment. In patients scheduled for PDS, primary tumour and metastasis biopsies were obtained during debulking surgery. In patients scheduled for NACT+IDS, metastasis biopsies were obtained during diagnostic laparoscopy (LPS). For immunohistochemical (IHC) analysis, formalin-fixed paraffin-embedded (FFPE) tissue blocks related to the diagnosis were collected and cut into 3–4 μM sections and mounted on superfrost slides. All slides were evaluated by a dedicated gynaeco-pathologist, blinded for patient characteristics. 

Haematoxylin and eosin (HE) staining of FFPE tissue sections was evaluated to determine the percentage of tumour cells (% TCs), assess morphologic tumour heterogeneity, and localise and estimate the percentage of tumour-associated immune cells (% immune cells present, ICP).

The percentage of TILs on HE-stained sections was then evaluated according to recommendations provided by the International TILs Working Group 2014 for breast cancer [53].

FFPE tissue sections were analysed for PD-L1 expression by IHC using the Ventana SP263 assay with OptiView DAB IHC Detection Kit on a VENTANA BenchMark ULTRA instrument, according to the manufacturer’s instructions. Counter-staining was performed as part of the automated staining protocol using haematoxylin. After staining, slides were washed in distilled water, dehydrated in graded alcohol and xylene, and mounted with a cover slip.

PD-L1 status was determined on TCs as the percentage of TCs with any membrane staining above background and on ICPs as the percentage of ICPs exhibiting PD-L1 staining of membrane, cytoplasm, or punctate staining at any intensity above background. In addition, PD-L1 status was categorised in each sample using a binary cut-off. Briefly, TCs were classified as positive if TCs ≥ 1% expressed PD-L1 (PD-L1^+^ TCs). Similarly, ICPs were classified positive if ICPs ≥ 1% expressed PD-L1 (PD-L1^+^ ICP).

### 4.3. Plasma Samples

Peripheral blood samples were obtained from HGSOC patients at primary diagnosis. Plasma was obtained by double centrifugation of citrated blood at 1800× *g* for 15 min, immediately frozen at −80 °C, and then transferred in liquid nitrogen for long storage. For the assay, plasma samples were thawed and diluted v/v with 100 nM filtered phosphate buffered saline (PBS).

### 4.4. Flow Cytometry Measuring of MVs in Patients’ Plasma

The Minimal Information for Studies of Extracellular Vesicles (MISEV) guidelines [54] were taken into account, and special attention was paid to blood collection, plasma centrifugation conditions, freezing methods, and sample staining, which could affect MV quantification. The MV staining procedure is detailed in the Supplementary Material S1. MVs were analysed on a CytoFLEX LX (Beckman Coulter, Brea, CA, USA). cytometer equipped with violet (405 nM), blue (488 nM), yellow-green (561 nM), and red (638 nM) laser excitation sources. The CytoFLEX LX is able to collect side scatter (SSC) off the violet laser (VSSC), and this served to improve MV resolution, as the 405 nM wavelength of the violet laser is closer to the MVs’ size than the most commonly used blue laser (488 nM) [55,56]. CytExpert software (Beckman Coulter) was used for acquisition and data extraction. Sample fluid stream size was kept at a minimum using the lowest flow rate featured on the instrument (10 μL/min) to improve hydrodynamic focusing—thereby optimising signal collection―and to reduce swarming recognition [56]. MV concentration was calculated using the volumetric measurement featured in the CytoFLEX LX. Flow cytometry data were independently analysed by two expert flow cytometrists in a single session (single batch analysis).

### 4.5. Statistical Analysis

Results are presented as absolute frequency (percentage) and as median (min–max) or mean (standard deviation), as appropriate. The normality of distribution of continuous variables was assessed with the Shaphiro–Wilks test. The Mann–Whitney test and χ^2^ or Fisher’s exact were used as appropriate to assess whether there were differences in the parameters of interest in patients (a) who underwent PDS versus those who underwent diagnostic LPS; (b) who had PD-L1^−^ (<1%) versus those who had PD-L1^+^ (≥1%) TCs and ICPs at histology; and (c) with FIGO stage IIIB–IIIC versus those with FIGO stage IVA–IVB [35]. All analyses were performed on the overall study population. Additional investigations were applied to those patients with both primary and metastatic tumour biopsies. The Spearman test was applied to investigate a possible correlation between the PD-L1^+^ TC percentage in the primary tumour and in the metastatic lesion. Two-sided tests were applied, and the significance level was set at *p* < 0.05. No imputation was carried out for missing data. STATA software (STATA/IC 16.0 for Windows, College Station, TX, StataCorp LP, 4905 Lakeway Drive, College Station, TX 77845, USA) was used for the analysis. GraphPAD Prism software (version 9.0.0 for Windows, San Diego, CA, USA) was used to plot data.

## 5. Conclusions

We propose a first-in-class flow cytometry approach for rapid detection of circulating TC- and leuko-derived PD-L1^+^ MVs in HGSOC patients. This test is minimally invasive, can be performed at any time point during disease course, and has, therefore, the potential to be inserted in algorithms for complementing IHC in assessing tumour PD-L1 status for a more efficient clinical management of OC patients in the era of precision medicine. The clinical relevance of circulating PLT-derived PD-L1^+^ MVs in HGSOC patients warrants further studies.

## Figures and Tables

**Table 1:** Clinical and pathological characteristics of the study population.

Characteristics	*n* (%)
Number of patients	63
Median age (min–max), years	59 (39–81)
Chemotherapy regimen	
Carboplatin Gemcitabine Bevacizumab	1 (1.6)
Carboplatin Taxol	31 (49.2)
Carboplatin Taxol Bevacizumab	1 (1.6)
Experimental protocol	30 (47.6)
ENGOT OV 43	13/30 (43.3)
MITO 28	1/30 (3.3)
AGO DUO	16/30 (53.3)
Clinical setting	
PDS+ACT	26 (41.3)
LPS+NACT	37 (58.7)
Histotype	
Serous carcinoma	62 (98.4)
Mixed	1 (1.6)
Grading	
1	0 (0)
2	0 (0)
3	63 (100)
FIGO stage	
IIIB	3 (4.8)
IIIC	45 (71.4)
IVA	4 (6.3)
IVB	11 (17.5)

Results are presented as n (%) except where indicated. PDS: Primary debulking surgery. ACT: Adjuvant chemotherapy. LPS: Laparoscopic surgery. FIGO: International Federation of Gynaecology and Obstetrics [35].

**Table 2:** PD-L1 analysis at histology according to the type of surgery.

Characteristics	All cases *n* = 63	PDS *n* = 26	Laparoscopic diagnostic *n* = 37	*p*-Value
PD-L1 analysis on ovarian sample				
Number of cases evaluated	26	26	0	
Specimen dimension, mm	15.5 (1–30)	15.5 (1–30)	-	-
TCs				-
<1%	13 (50.0)	13 (50.0)	-	
≥1%	13 (50.0)	13 (50.0)	-	
% expression, median (min–max)	1 (0–30)	1 (0–30)	-	-
ICP				-
<1%	18 (69.2)	18 (69.2)	-	
≥1%	8 (30.8)	8 (30.8)	-	
PD-L1 analysis on metastatic sample				
Number of cases evaluated	63	26	37	
Specimen dimension, mM	7 (1–24)	9.5 (1–24)	5 (1–20)	
TCs				0.161
<1%	38 (60.3)	13 (50.0)	25 (67.6)	
≥1%	25 (39.7)	13 (50.0)	12 (32.4)	
% expression, median (min–max)	1 (0–85)	1 (0–30)	1 (0–85)	0.456
ICP				0.091
<1%	48 (76.2)	17 (65.4)	31 (83.8)	
≥1%	15 (23.8)	9 (34.6)	6 (16.2)	
Parietal peritoneum				
Number of cases evaluated	33	10	23	
TCs				0.520
<1%	16 (48.5)	4 (40.0)	12 (52.2)	
≥1%	17 (51.5)	6 (60.0)	11 (47.8)	
ICP				0.164
<1%	25 (75.8)	6 (60.0)	19 (82.6)	
≥1%	8 (24.2)	4 (40.0)	4 (17.4)	
Pelvic peritoneum				
Number of cases evaluated	9	4	5	
TCs				0.444
<1%	8 (88.9)	3 (75.0)	5 (100)	
≥1%	1 (11.1)	1 (25.0)	0 (0)	
ICP				
<1%	7 (77.8)	2 (50.0)	5 (100)	0.167
≥1%	2 (22.2)	2 (50.0)	0 (0)	
Diaphragmatic peritoneum				
Number of cases evaluated	15	6	9	
TCs				0.235
<1%	11 (73.3)	3 (50.0)	8 (88.9)	
≥1%	4 (26.7)	3 (50.0)	1 (11.1)	
ICP				1
<1%	12 (80.0)	5 (83.3)	7 (77.8)	
≥1%	3 (20.0)	1 (16.7)	2 (22.2)	
Omentum				
Number of cases evaluated	6	6	0	
TCs				-
<1%	3 (50.0)	3 (50.0)	-	
≥1%	3 (50.0)	3 (50.0)	-	
ICP				-
<1%	4 (66.7)	4 (66.7)	-	
≥1%	2 (33.3)	2 (33.3)	-	
PD-L1 overall assessment				
Number of cases evaluated	63	26	37	
TCs				0.423
<1%	28 (44.4)	10 (38.5)	18 (48.6)	
≥1%	35 (55.6)	16 (61.5)	19 (51.4)	
ICP				0.872
<1%	44 (69.8)	13 (50.0)	31 (83.8)	
≥1%	19 (30.2)	13 (50.0)	6 (16.2)	

Results are presented as *n* (%) except where indicated. PD-L1: programmed cell death protein-1 ligand. TCs: tumour cells. ICP: immune cells present. PDS: primary debulking surgery.

**Table 3:** PD-L1 evaluation in patients with both ovarian cancer and metastatic biopsies.

Characteristic *	PD-L1 Negative in Ovary	PD-L1 Positive in Ovary	*p*-Value
TCs	-	-	0.006
PD-L1 negative in metastatic biopsies	10 (76.9)	3 (23.1)	-
PD-L1 positive in metastatic biopsies	3 (23.1)	10 (76.9)	-
ICP	-	-	0.272
PD-L1 negative in metastatic biopsies	13 (72.2)	4 (50.0)	-
PD-L1 positive in metastatic biopsies	5 (27.8.)	4 (50.0)	-

Results are presented as *n* (%). Bold font highlights statistically significant difference. TC: tumour cells. ICP: immune cells present. PD-L1: programmed cell death protein-1 ligand. * PD-L1 was considered positive and negative in cases of PD-L1+ ≥ 1% and PD-L1+ < 1% cells, respectively.

**Table 4:** Plasma concentration of microvesicles of different origin.

Characteristics	All Cases *n* = 63	FIGO Stage IIIB–IIIC *n* = 48	FIGO Stage IVA–IVB *n* = 15	*p*-Value
TC-derived microvesicles	109.7 (23.9–2560.4)	121.2 (23.9–2560.4)	92.5 (56.4–2332.9)	1
PD-L1^+^ TC-derived microvesicles	10.5 (1.4–178.8)	11.1 (1.4–178.8)	7.6 (2.4–46.4)	0.550
Leuko-derived microvesicles	126.2 (32.5–773.2)	132.3 (41.1–773.2)	112.8 (32.5–211.5)	0.287
PD-L1^+^ leuko-derived microvesicles	18.4 (6.2–504.8)	18.5 (6.2–504.8)	18.4 (6.5–54.5)	0.473
PLT-derived microvesicles	16084.7 (1171.1–143256.6)	14268.3 (1171.1–143256.6)	17327.5 (5640.4–122559.2)	0.325
PD-L1^+^ PLT-derived microvesicles	104.2 (10.3–2409.6)	100.5 (10.3–2409.6)	110.7 (20.8–615.2)	0.463

Results are presented as median (min–max). FIGO: International Federation of Gynaecology and Obstetrics [23]. TC: tumour cells. PD-L1: Programmed cell death protein-1 ligand. PLT: platelet.

## Data Availability

The datasets used and/or analysed during the present study are available from the corresponding author upon reasonable request.

## Supplementary Materials

The following are available online at https://www.mdpi.com/article/10.3390/cancers13205200/s1, Supplementary Material S1: Microvesicle Staining.
