# Supplementary material for: PD-L1 Expression on Circulating Tumour-Derived Microvesicles as a Complementary Tool for Stratification of High-Grade Serous Ovarian Cancer Patients
Source: Cancers (Basel). 2021 Oct 16;13(20):5200. doi: 10.3390/cancers13205200 (PMC8534085; doi:10.3390/cancers13205200)
Supplement: Supplementary file 1 [file cancers-13-05200-s001.zip › cancers-1413261-supplementary.pdf]

cancers

MDPI

Supplementary Materials

PD-L1 Expression on Circulating Tumour-Derived Microvesicles as A Complementary Tool for Stratification of High-Grade Serous Ovarian Cancer Patients

Alessandra Battaglia, Alessia Piermattei, Alexia Buzzonetti, Tina Pasciuto, Nicole Zampetti, Marco Fossati, Giuseppe Angelico, Valentina Iacobelli, Camilla Nero, Veronica Iannucci, Giovanni Scambia, Anna Fagotti and Andrea Fattorossi

Citation: Battaglia, A.; Piermattei, A.; Buzzonetti, A.; Pasciuto, T.; Zampetti, N.; Fossati, M.; Angelico, G.; Iacobelli, V.; Nero, C.; Iannucci, V.; et al. PD-L1 Expression on Circulating Tumour-Derived Microvesicles as A Complementary Tool for Stratification of High-Grade Serous Ovarian Cancer Patients. *Cancers* **2021**, *13*, 5200. https://doi.org/10.3390/cancers13205200

Academic Editors: Daniela Gallo, Claudia Marchetti and Anders Jakobsen

Received: 23 September 2021
Accepted: 14 October 2021
Published: 16 October 2021

Publisher's Note: MDPI stays neutral with regard to jurisdictional claims in published maps and institutional affiliations.

Copyright: © 2021 by the authors. Licensee MDPI, Basel, Switzerland. This article is an open access article distributed under the terms and conditions of the Creative Commons Attribution (CC BY) license (http://creativecommons.org/licenses/by/4.0/).

Materials S1. Microvesicle Staining

In the present study, we used EpCAM—antigen usually overexpressed by tumours of epithelial origin—to identify the TC-derived MVs and the common leukocyte antigen CD45 to identify Leuko-derived MVs. Phalloidin-FITC, the non-ionic detergent Triton X-100 and monoclonal antibodies (MoAbs) to CD326-PE (EpCAM, clone 1B7) were purchased from ThermoFisher; MoAb to CD274-BV421 (PDL-1, clone MIH1) was purchased from BD Biosciences, MoAbs to CD45-KO (clone HI30) and to CD41a-ECD (clone P2) were purchased from Beckman Coulter. Appropriate isotype controls were used. Phalloidin-FITC (Sigma) was used to identify non-integer microvesicles (MVs). Phalloidin binds with high selectivity F-actin, an internal molecule, and non-specific staining is negligible. Phalloidin-FITC (1:200 final dilution) was added to pre-mixed, pre-optimized MoAb cocktail in all experiments.

Tumour cell (TC)-derived MVs were identified as MVs staining positively for EpCAM. Leukocyte (Leuko)-derived MVs were identified as MVs staining positively for CD45. Signals of PE, BV421 and KO required minimal compensation and generated marginal spreading error as these fluorochromes have little to no spectral overlap with each other.

The MoAb/Phalloidin combination (95 µL) or appropriate isotype control/Phalloidin combination were mixed with 100 µL plasma that had been previously diluted 1:2 in 100 nm-filtered PBS. Being impossible to titrate correctly MoAbs on MVs, all MoAbs were used at a final dilution of 1:40. To reduce false positive events from MoAb aggregates, the MoAbs/Phalloidin mixture was centrifuged using a fixed-angle single speed centrifuge (~18,000× *g*) at RT for 15 min immediately before use. To check signal from MoAb aggregates that survived centrifugation, each staining session included the MoAb/Phalloidin mixture in the absence of plasma and plasma in the absence of the MoAb/Phalloidin mixture. Incubation of plasma with MoAb/Phalloidin mixture lasted 45 min at RT. Before the flow cytometry run, plasma samples were diluted with 1ml of 100 nm-filtered PBS to minimize coincidences (swarming recognition) and reduce fluorescence signal out of unbound MoAbs.

As additional staining control, samples were run after MVs membrane had been disrupted with 1% Triton-X-100 to verify disappearance of positive events [1]. Exposure to the detergent caused almost complete disappearance of PD-L1+EpCAM+ and PD-L1+CD45+ events, thus confirming their tridimensional structure and sensitivity to digestion, typical of MVs [1].

The set-up of the instrument, a critical point for MVs analysis, has been detailed before [2]. Briefly, the best gains for the photodiodes detecting fluorescence and VSSC-A signals were determined using the 8-Peak Rainbow Beads and Megamix-Plus FSC beads, respectively. The Megamix-Plus FSC beads served also to establish the window of analysis, i.e., the MVs region, in a dual colour VSSC-A/fluorescence plot. For the present work, the threshold was set on the VSSC-A signal of Megamix-Plus FSC beads and was

further refined to include events producing a VSSC-A signal slightly lower than that generated by the smallest detectable PLT-derived MVs. This was the best compromise between good resolution and a level of background noise that did not impede cytometer performance: lowering the VSSC-A signal threshold further produced an excess of background signal.

To take into account the dilution factor consequent to sample treatment and staining, the final concentration of MVs in plasma samples was calculated multiplying the MVs concentration in the test tube by 23.9 (final dilution of the plasma sample in the test tube). Quality control of flow cytometry was performed on each experimental day. Flow cytometer configuration and settings were never changed. To minimize differences in sample staining, the same reagents' batches were used throughout the study and all plasma samples were thawed, processed and run within 3 h from thawing. Moreover, a batch processing strategy was used: plasma samples were processed in 7 flow cytometry acquisition sessions over 17 days. The MoAb/Phalloidin mixture sufficient for the staining of plasma samples was freshly prepared on each experimental day. Cytometry run was stopped after 10 min record time or when 3 × 106 events were acquired, whichever came first (minimum number of acquired events 238,885). Abort rate never exceeded 6.2% (minimum 2.7%; with a median of 3.5%).

Reference

1. Cha, J.M.; Shin, E.K.; Sung, J.H.; Moon, G.J.; Kim, E.H.; Cho, Y.H.; Park, H.D.; Bae, H.; Kim, J.; Bang, O.Y.; et al. Efficient scalable production of therapeutic microvesicles derived from human mesenchymal stem cells. *Sci. Rep.* **2018**, *8*, 1171.
2. Lucchetti, D.; Battaglia, A.; Ricciardi-Tenore, C.; Colella, F.; Perelli, L.; de Maria, R.; Scambia, G.; Sgambato, A.; Fattorossi, A. Measuring Extracellular Vesicles by Conventional Flow Cytometry: Dream or Reality? *Int. J. Mol. Sci.* **2020**, *21*, 6257, doi:10.3390/ijms21176257.
